# Immunofluorescence study of cytoskeleton in endothelial cells induced with malaria sera

**DOI:** 10.1186/s12936-023-04833-7

**Published:** 2024-01-05

**Authors:** Mathusorn Wongsawat, Supattra Glaharn, Charit Srisook, Wilanee Dechkhajorn, Urai Chaisri, Chuchard Punsawad, Tachpon Techarang, Kesinee Chotivanich, Srivicha Krudsood, Parnpen Viriyavejakul

**Affiliations:** 1 Department of Tropical Pathology, Faculty of Tropical Medicine, Mahidol University, 420/6 Rajvithi Road, Bangkok, 10400 Thailand; 2 Department of Medical Science, School of Medicine, Walailak University, Nakhon Si Thammarat, 80160 Thailand; 3 Research Center in Tropical Pathobiology, Walailak University, Nakhon Si Thammarat, 80160 Thailand; 4 Department of Clinical Tropical Medicine, Faculty of Tropical Medicine, Mahidol University, 420/6 Rajvithi Road, Bangkok, 10400 Thailand; 5 Department of Tropical Hygiene, Faculty of Tropical Medicine, Mahidol University, 420/6 Rajvithi Road, Bangkok, 10400 Thailand

**Keywords:** Malaria, *Plasmodium falciparum*, Cytoskeleton, Actin, Tubulin, Vimentin, Endothelial cells, Immunofluorescence, TNF, IFN-γ

## Abstract

**Background:**

Endothelial cells (ECs) play a major role in malaria pathogenesis, as a point of direct contact of parasitized red blood cells to the blood vessel wall. The study of cytoskeleton structures of ECs, whose main functions are to maintain shape and provide strength to the EC membrane is important in determining the severe sequelae of *Plasmodium falciparum* malaria. The work investigated the cytoskeletal changes (microfilaments-actin, microtubules-tubulin and intermediate filaments-vimentin) in ECs induced by malaria sera (*Plasmodium vivax*, uncomplicated *P. falciparum* and complicated *P. falciparum*), in relation to the levels of pro-inflammatory cytokines.

**Methods:**

Morphology and fluorescence intensity of EC cytoskeleton stimulated with malaria sera were evaluated using immunofluorescence technique. Levels of tumour necrosis factor (TNF) and interferon (IFN)-gamma (γ) were determined using enzyme-linked immunosorbent assay (ELISA). Control experimental groups included ECs incubated with media alone and non-malaria patient sera. Experimental groups consisted of ECs incubated with malaria sera from *P. vivax*, uncomplicated *P. falciparum* and complicated *P. falciparum*. Morphological scores of cytoskeletal alterations and fluorescence intensity were compared across each experiment group, and correlated with TNF and IFN-γ.

**Results:**

The four morphological changes of cytoskeleton included (1) shrinkage of cytoskeleton and ECs with cortical condensation, (2) appearance of eccentric nuclei, (3) presence of “spiking pattern” of cytoskeleton and EC membrane, and (4) fragmentation and discontinuity of cytoskeleton and ECs. Significant damages were noted in actin filaments compared to tubulin and vimentin filaments in ECs stimulated with sera from complicated *P. falciparum* malaria. Morphological damages to cytoskeleton was positively correlated with fluorescence intensity and the levels of TNF and IFN-γ.

**Conclusions:**

ECs stimulated with sera from complicated *P. falciparum* malaria showed cytoskeletal alterations and increased in fluorescence intensity, which was associated with high levels of TNF and IFN-γ. Cytoskeletal changes of ECs incubated with complicated *P. falciparum* malaria sera can lead to EC junctional alteration and permeability changes, which is mediated through apoptotic pathway. The findings can serve as a basis to explore measures to strengthen EC cytoskeleton and alleviate severe malaria complications such as pulmonary oedema and cerebral malaria. In addition, immunofluorescence intensity of cytoskeleton could be investigated as potential prognostic indicator for malaria severity.

## Background

Malaria is a life-threatening disease which remains endemic in subtropical and tropical regions [1]. Among the five species of human malaria, *Plasmodium falciparum* is the most malignant form causing severe complications, such as cerebral malaria, pulmonary oedema, acute kidney injury and severe anaemia [2]. Important pathogenesis of severe malaria involves the integration of two main essential mechanisms, namely mechanical and chemical processes. Mechanical process of cytoadherence between parasitized red blood cells (PRBCs) and endothelial cells (ECs) leads to sequestration of PRBCs into the microvasculature [3], which causes occlusion of blood vessels in the internal organs. During cytoadherence process, PRBCs adhere to the ECs via parasite-derived proteins expressed on the surface of the PRBCs. The knob-mediated cytoadherence to specific EC receptors initiates the severe sequelae of complicated *P. falciparum* malaria [4] and has been linked to pro-inflammatory induction [5]. Chemical process comprises the release of soluble cytokines, such as tumour necrosis factor (TNF), interleukins (IL) [6, 7], and interferon gamma (IFN-γ) [8] from activated monocytes and macrophages during malaria infection. Subsequent activation of the host immune responses to malaria causes severe clinical manifestations and pathological effects. As ECs are vital to malaria pathogenesis and disease progression, studies of post-adhesive signaling events are essential in identifying changes of ECs during malaria infection. Actin is the most abundant intracellular protein in the cytoplasm of eukaryotic cells [9] and an important component of the cytoskeleton, as a dynamic cell structure in various cell types. Actin filaments exists as two forms, namely monomeric globular actin called G-actin and filamentous actin called F-actin, formed by G-actin polymerization [9]. The microtubules (polymers of tubulin) are considered principal component of the cytoskeleton. They are rigid hollow rods, approximately 25 nm in diameter and play important role in organizing organelles [10]. The dynamic structures of tubulin filaments undergo continual assembly and disassembly within the cell. Tubulin filaments determines cell shape and cell movements, including forms of cell locomotion and the separation of chromosomes during mitosis, essentially for the intracellular transport of organelles [11]. Intermediate filaments are composed of a variety of proteins that are expressed in different types of cells. In ECs, intermediate filaments are expressed as vimentin, typically known as type III intermediate filament protein [12]. Vimentin filaments is also the key component in maintaining the overall integrity of cytoplasm. The functions of which involve supporting ECs and cytoskeletal integrities as well as cell shape, similar to the actin and tubulin filaments [13].

Studies have shown that pro-inflammatory cytokines, such as TNF, IL-1 and IFN-γ cause alterations in EC permeability in malaria [7, 24], but no extensive study of cytoskeletal changes of ECs in post-malaria infection and the association with pro-inflammatory cytokines have been reported. The morphological study of cytoskeleton in ECs induced by malaria sera could delineate the damage occur during malaria infection and could be a basis for strengthening EC cytoskeleton as an important cellular barrier and possibly prevent malaria complications.

## Methods

### Specimen preparation

Blood specimens were from the left-over sera of non-malaria patients (control), *Plasmodium vivax* and *P. falciparum* malaria patients (both uncomplicated and complicated *P. falciparum* malaria). The specimens were stored at the Department of Tropical Pathology, Faculty of Tropical Medicine, Mahidol University, during the period of 2016–2022. Specimens were divided into 5 groups (5 isolates per group), namely sera from *P. vivax*, uncomplicated *P. falciparum*, complicated *P. falciparum*, and two control groups consisted of media alone and sera from non-malaria patients (as negative control). Inclusion criteria for malaria patients were a positive malaria parasite on blood film examination, male or female patients with age between 18 and 60 years old, with no other serious underlying diseases such as diabetes mellitus or a co-infection with bacteria or fungus.

Classification for complicated *P. falciparum* malaria was in accordance with World Health Organization (WHO) criteria [2]. Non-malaria patient sera were from volunteers aged 18–60 years of any gender with no serious underlying diseases and no previous infection to malaria. Informed consent was obtained from all patients. The study protocol was approved by the Ethics Committee, Faculty of Tropical Medicine, Mahidol University (MUTM 2021-032-01 and MUTM 2021-032-02).

### Co-culture of HUVECs with malaria sera

Human umbilical vein endothelial cells (HUVECs) (PromoCell, Heidelberg, Germany) were grown in 25 cm^2^ flasks, coated by 1% gelatin solution according to the manufacturer’s instructions. The fourth to sixth passages were used for the experiments. Co-culture of HUVECs with malaria sera was performed based on previous study with minor modifications [14]. ECs were grown on an 8-well cell culture slide (SPL Life Sciences Co., Ltd., Gyeonggi-do, Korea) until confluence. When cells reach > 90% confluence, complete EC media was removed and replaced with new serum-free media containing malaria sera (10%) from *P. vivax*, uncomplicated *P. falciparum* and complicated *P. falciparum*. Control groups included incubation of HUVECs with serum-free media alone and serum-free media mixed with non-malaria patient sera (10%). Incubation periods were at specific times (T) of 0, 30, 60 and 90 min.

### Measurement of tumour necrosis factor and interferon-γ levels

The levels of TNF and IFN-γ in malaria sera were determined from sera obtained from the day of admission. Pro-inflammatory cytokines were analysed by enzyme-linked immunosorbent assay (ELISA) kits [BD OptEIA^™^ Human TNF ELISA Kit II (BD Biosciences, Oxford, UK) and Human IFN-γ Mini TMB ELISA Development Kit (PeproTech, NJ, USA)]. Assays were performed according to the manufacturer’s instructions. Briefly, 100 µl of standard and samples were added in the pre-coated 96-well plate for 2 h at room temperature. Either a biotinylated purified rabbit anti-human TNF or IFN-γ was used to detect specific antibodies. An avidin-horseradish peroxidase (HRP) conjugate was added, followed by 3, 3’, 5, 5’-Tetramethylbenzidine (TMB) substrate for colorimetric detection. Finally, the reaction was stopped with a commercial stop solution to block the TMB oxidized by HRP. Optical density was read at 450 nm with a microtiter plate reader (Azure Biosystems Inc, CA, USA). All samples were measured in triplicate. The minimum detectable dose of TNF was determined to be 2 pg/ml and the sensitivity for IFN-γ was within the range of 16–2000 pg/ml, based on the manufacturer’s protocols.

### Immunofluorescence study of actin, tubulin and vimentin filaments

The investigation of EC cytoskeleton components was performed by using immunofluorescence technique. Direct immunofluorescence technique was applied to investigate the structures of actin filaments (both of F-actin and G-actin), and indirect immunofluorescence technique was used to examine the frameworks of microtubules (tubulin) and intermediate filaments (vimentin), following previous study with modifications [15]. After incubation, malaria sera were removed at specific time points and HUVECs were washed by 1X PBS (3 times). The ECs were fixed with 4% paraformaldehyde for 15 min, washed in 1X PBS (3 times for 10 min) and permeabilized with Triton X-100 (0.1% solution in 1X PBS) for 15 min. To reduce non-specific background staining, ECs were treated with blocking buffer (5% BSA solution in 1X PBS plus 0.25% Triton X-100 in 1X PBS for F- and G-actin and 10% normal goat serum for 30 min for tubulin and vimentin filaments).

For F- and G-actin staining using direct immunofluorescence, ECs were labelled with specific primary antibodies (phalloidin for F-actin and Deoxyribonuclease I for G-actin conjugated with Alexa Fluor 594 and 488, respectively) (F- and G-actin: 1:200 dilution; Invitrogen, Waltham, MA, USA). Indirect immunofluorescence technique was performed by using β-tubulin (1:100 dilution; Bioss, USA) and vimentin (1:100 dilution; Santa Cruz Biotechnology, Inc., USA) as primary antibodies. Secondary antibodies included Alexa Fluor 488 (1:200 dilution; Abcam, USA) for β-tubulin and Alexa Fluor 594 (1:200 dilution; Abcam, USA) for vimentin. Incubation time was 1 h, at 37 °C, then cells were washed in 1X PBS (3 times) and finally slides were mounted with ProLong^™^ Gold antifade reagent mixed with DAPI (Invitrogen, Waltham, MA, USA). Morphological changes of actin, tubulin, vimentin and EC components were evaluated using laser scanning confocal microscope (LSM700, Carl Zeiss AG, Germany).

### Evaluation of EC cytoskeleton and EC morphology

Cytoskeleton changes and cell morphology of ECs were evaluated based on aberrations of cytoskeletal filaments and appearance of ECs after incubation with malaria sera. Each experimental group was evaluated in 15 microscopic fields (> 300 cells), under low power magnification (200X) and expressed as percentage. Percentage of morphological changes was calculated based on the number of cytoskeletal alterations to the total number of cells counted, multiplied by 100. Mean morphological scores were compared across each experiment groups by summation of each morphological changes. For immunofluorescence intensity, mean intensity was evaluated on two hundred ECs per experimental group and analysed by ZEN 2012 (blue edition) software program (Carl Zeiss AG, Germany).

### Statistical analysis

The results were expressed as mean ± standard error of the mean (SEM). All quantitative data were tested for normality of distribution by Kolmogorov–Smirnov test. The non-parametric data were analysed by Kruskal–Wallis test (H test) and Mann Whitney *U* test. Pro-inflammatory cytokine levels were analysed by one-way analysis of variance (ANOVA). Difference between experimental groups was evaluated by Fisher’s least significant difference (LSD) method. The correlations between the morphological scores of cytoskeleton alterations, fluorescence intensity and levels of pro-inflammatory cytokines were analysed by Spearman’s rank correlation coefficient (*r*_*s*_). Statistical analysis was performed by using PASW Statistics 18 (formerly SPSS, International Business Machines, IL, USA). The results were considered statistically significant at the 95% confidence interval (*p* < 0.05).

## Results

### Morphological changes of cytoskeleton stimulated with malaria sera

#### Actin filaments

**Fig. 1:**
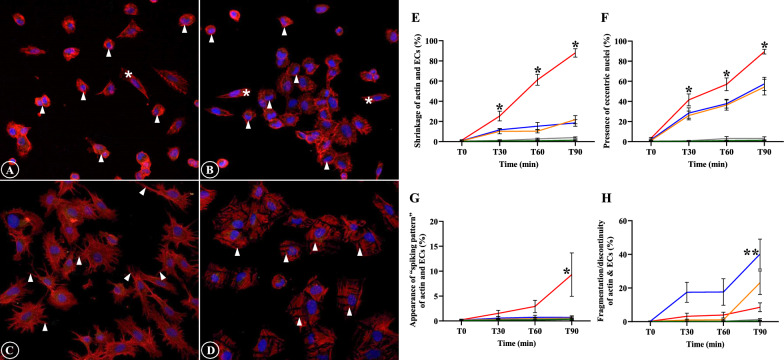
The alterations of actin filaments and ECs induced by malaria sera. **A** and **E**: Shrinkage of actin filaments and ECs with cortical condensation (asterisk- normal ECs, arrowheads- shrinkage); **B** and **F**: Appearance of eccentric nuclei (asterisks- normal ECs, arrowheads- eccentric nuclei); **C** and **G**: Presence of “spiking pattern” of actin filaments and EC membrane (arrowheads); **D** and **H**: Fragmentation and discontinuity of actin filaments and ECs (arrowheads). **A**–**D**: ECs induced by sera from complicated *P. falciparum* (magnification X200). ECs were incubated with media only (green line), non-malaria patient sera (grey line), malaria sera from *P. vivax* (orange line), uncomplicated *P. falciparum* (blue line) and complicated *P. falciparum* (red line). *Significant difference of complicated *P. falciparum* compared with all experimental groups (*p* < 0.05). **Significant difference of uncomplicated *P. falciparum* compared with all experimental groups (*p* < 0.05). Data are presented as mean ± SEM

**Fig. 2:**
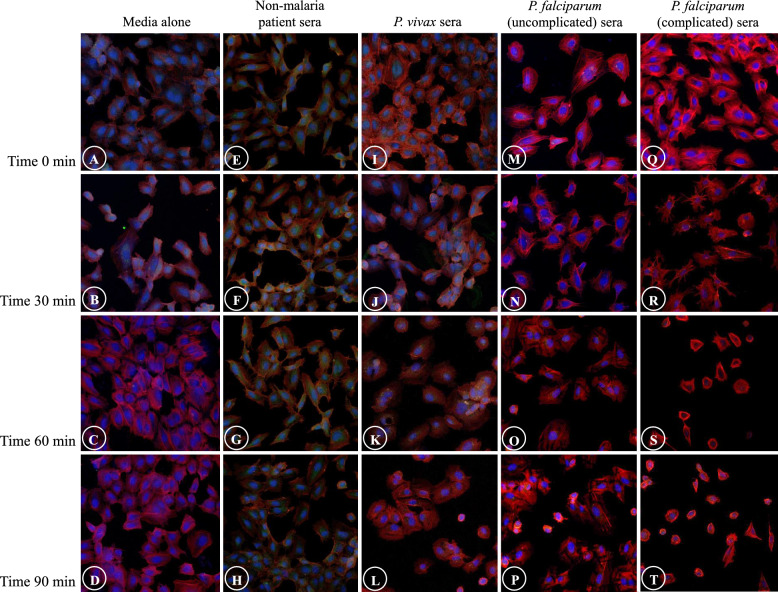
Comparative morphological alterations of actin filaments in different experimental groups at different time points. Severe morphological damages are mostly illustrated in ECs induced with sera from complicated *P. falciparum* malaria (last column). (magnification X200)

The alterations of filamentous actin and ECs included shrinkage of actin filaments and ECs, appearance of eccentric nuclei, presence of “spiking pattern” of actin filaments and EC membrane, in addition to the fragmentation and discontinuity of actin filaments and ECs. Actin filaments and ECs showed distinct contraction and morphologically reduced in cell size (Fig. 1A). Actin filament accumulation was frequently seen near the eccentric EC nuclei and nuclei appeared as peripherally located. (Fig. 1B). The “spiking pattern” of actin filaments and EC membrane appeared as “pointing out” of the filamentous actin and cell membrane (Fig. 1C). Actin filaments progressing to fragmentation can be described as cells undergoing disintegration and dissolution (Fig. 1D). For actin filaments and EC shrinkage, and presence of eccentric nuclei, prominent morphological changes were significantly highest in ECs incubation with sera from complicated *P. falciparum* malaria compared to all experimental groups from T30 min (*p* < 0.05) (Fig. 1E, F). Appearance of “spiking pattern” of actin filaments and EC membrane was significantly elevated at T90 in ECs incubation with sera from complicated *P. falciparum* malaria (Fig. 1G). Highest fragmentation and discontinuity of actin filaments and ECs were significantly observed in ECs induced with malaria sera from uncomplicated *P. falciparum* after 90 min (*p* < 0.05) (Fig. 1H). Figure 2 shows the morphological changes of actin filaments and ECs in different experimental groups at different time points.

#### Tubulin filaments

**Fig. 3:**
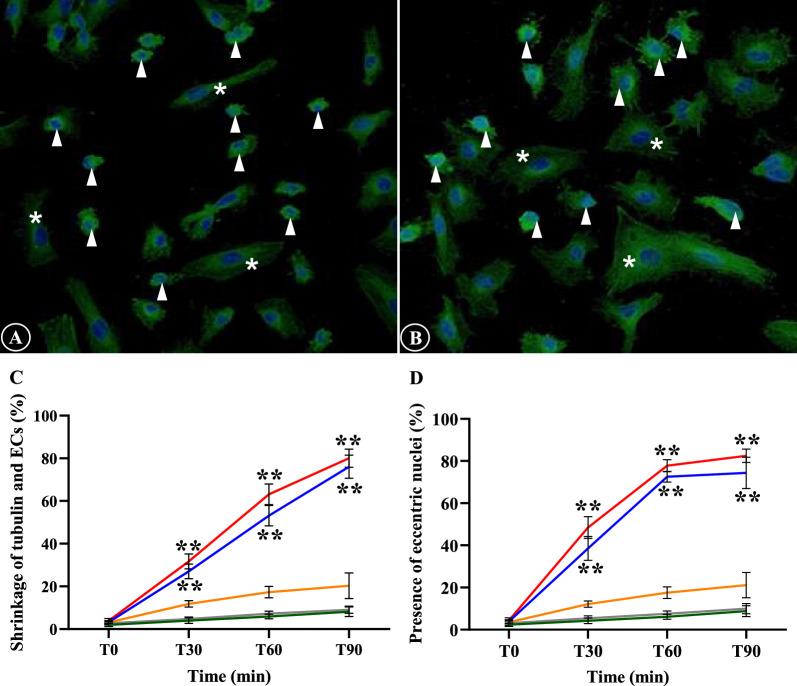
The morphology of tubulin filaments and EC induced by malaria sera. **A** and **C**: Shrinkage of tubulin filaments and ECs with cortical condensation (arrowheads); **B** and **D**: Appearance of eccentric nuclei (arrowheads). Normal ECs are shown in asterisks. **A**, **B**: ECs induced by sera from complicated *P. falciparum*. (magnification X200). ECs were incubated with media only (green line), non-malaria patient sera (grey line), malaria sera from *P. vivax* (orange line), uncomplicated *P. falciparum* (blue line) and complicated *P. falciparum* (red line). **Significant difference of *P. falciparum* compared with *P. vivax* and control groups (*p* < 0.05). Data are presented as mean ± SEM

**Fig. 4:**
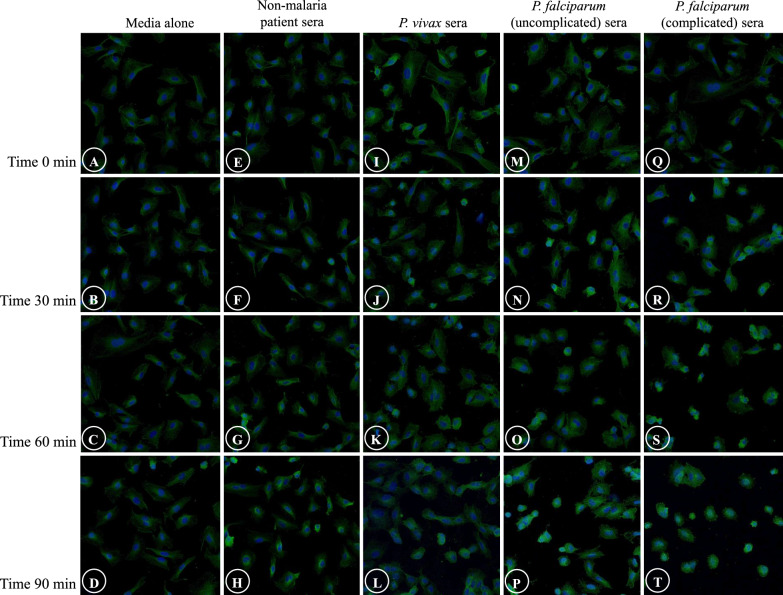
Comparative morphological alterations of tubulin filaments in different experimental groups at different time points. Shrinkage of tubulin filaments and ECs, and appearance of eccentric nuclei are mostly observed in ECs induced with sera from *P. falciparum* malaria. (magnification X200)

Tubulin filament disruption after stimulation with malaria sera included only shrinkage of tubulin filaments and ECs, and appearance of eccentric nuclei (Fig. 3A, B). There was a significant difference in shrinkage of tubulin filaments and ECs, and presence of eccentric nuclei of *P. falciparum* groups (both uncomplicated and complicated malaria) at T30, T60 and T90 min when compared to *P. vivax* and control groups (Fig. 3C, D). No difference was observed between complicated *P. falciparum* group and uncomplicated *P. falciparum* group at all the time (*p* > 0.05). Comparative morphological changes of tubulin filaments at different time points of different experimental groups are depicted in Fig. 4.

#### Vimentin intermediate filament

**Fig. 5:**
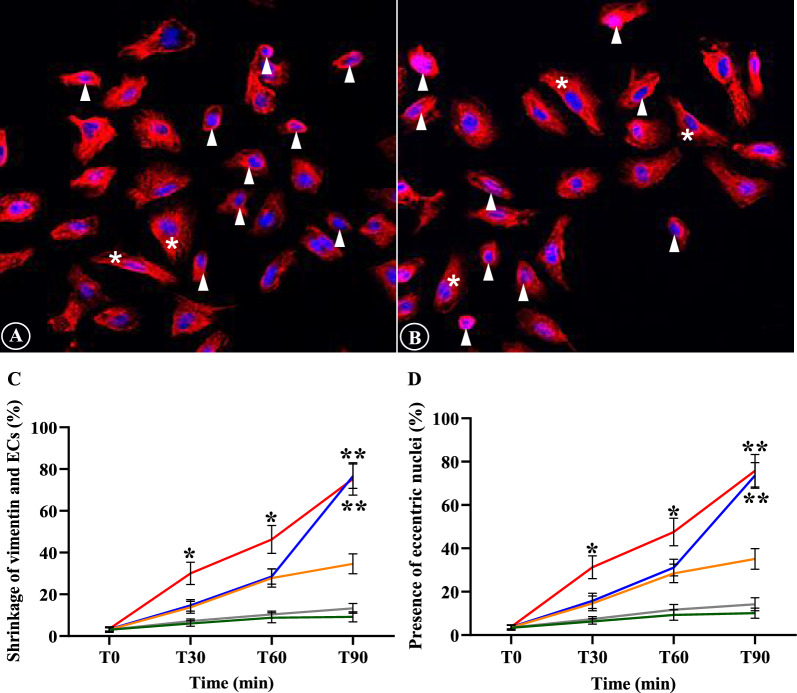
The morphology of vimentin filaments and ECs induced by malaria sera. **A** and **C**: Shrinkage of vimentin filaments and ECs with cortical condensation (arrowheads); **B** and **D**: Appearance of eccentric nuclei (arrowheads). Normal ECs are shown in asterisks. **A**, **B**: ECs induced by sera from complicated *P. falciparum*. (magnification X200). ECs were incubated with media only (green line), non-malaria patient sera (grey line), malaria sera from *P. vivax* (orange line), uncomplicated *P. falciparum* (blue line) and complicated *P. falciparum* (red line). *Significant difference of complicated *P. falciparum* compared with all experimental groups (*p* < 0.05). **Significant difference of *P. falciparum* compared with *P. vivax* and control groups (*p* < 0.05). Data are presented as mean ± SEM

**Fig. 6:**
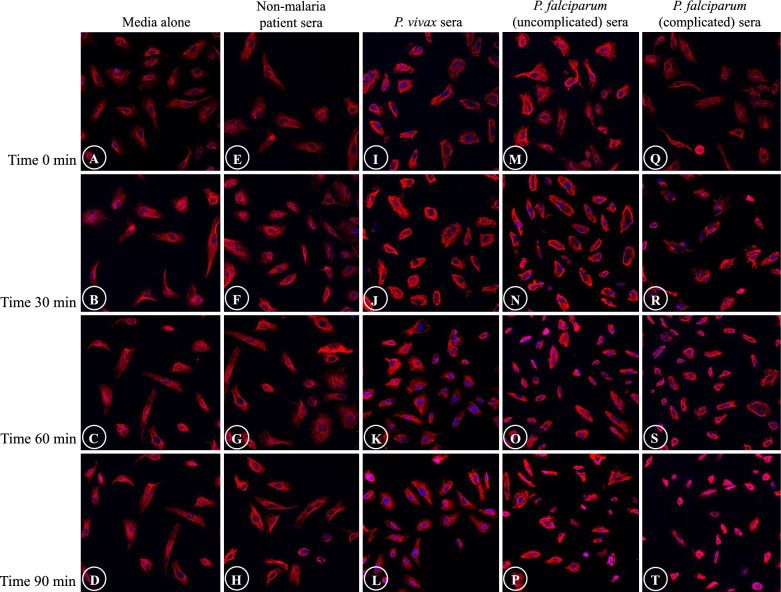
Comparative morphological alterations of vimentin filaments in different experimental groups at different time points. Shrinkage of vimentin filaments and ECs, and appearance of eccentric nuclei are mostly noted in ECs induced with sera from *P. falciparum* malaria at T90 min. (magnification X200)

Similar to tubulin filaments, only shrinkage of vimentin filaments and ECs, and presence of eccentric nuclei were observed in vimentin filaments induced by malaria sera (Fig. 5A, B). There was a significant difference in vimentin filaments and EC shrinkages (Fig. 5C) and the appearance of eccentric nuclei (Fig. 5D) of complicated *P. falciparum* group at T30 and T60 min when compared with all experimental groups (*p* < 0.05). At T90 min, vimentin filaments and EC changes showed no significant difference when compared with uncomplicated and complicated *P. falciparum* groups. Comparative morphological changes of vimentin filaments at different time points of different experimental groups are depicted in Fig. 6.

#### Overall morphological scores of cytoskeleton changes

**Fig. 7:**
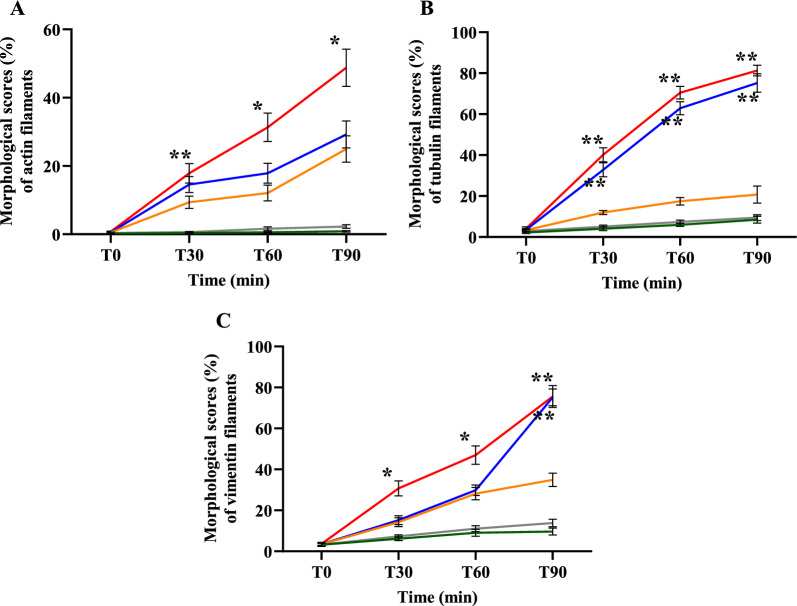
The overall morphological changes for cytoskeleton at different time points of different experimental groups. **A**: actin filaments; **B**: tubulin filaments and **C**: vimentin filaments. ECs were incubated with media only (green line), non-malaria patient sera (grey line), malaria sera from *P. vivax* (orange line), uncomplicated *P. falciparum* (blue line) and complicated *P. falciparum* (red line). *Significant difference of complicated *P. falciparum* compared with all experimental groups (*p* < 0.05). **Significant difference of *P. falciparum* compared with *P. vivax* and control groups (*p* < 0.05). Data are presented as mean ± SEM

The overall morphological scores for cytoskeleton alterations and ECs (shrinkage of cytoskeleton and ECs with cortical condensation, appearance of eccentric nuclei, presence of “spiking pattern” of cytoskeleton and EC membrane, and fragmentation and discontinuity of cytoskeleton and ECs) at different time points are illustrated in Fig. 7. The control groups (non-malaria patient sera and media alone groups) showed significantly lower overall morphological scores compared to malaria groups. At T60 min onwards, ECs incubated with sera from complicated *P. falciparum* malaria showed highest damage to actin filaments and ECs (*p* < 0.05) (Fig. 7A). For tubulin filaments, morphological scores demonstrated statistical differences between ECs incubated with sera from *P. falciparum* malaria groups and other experimental groups (*p* < 0.05) (Fig. 7B). In addition, the overall morphological scores of vimentin filaments showed significant difference at T30 and T60 min (*p* < 0.05) (Fig. 7C). No significant difference at T90 between ECs incubated with sera from complicated and uncomplicated *P. falciparum* malaria groups was observed.

### Fluorescence intensity of cytoskeleton accumulation in ECs induced with malaria sera

#### Actin filaments

**Fig. 8:**
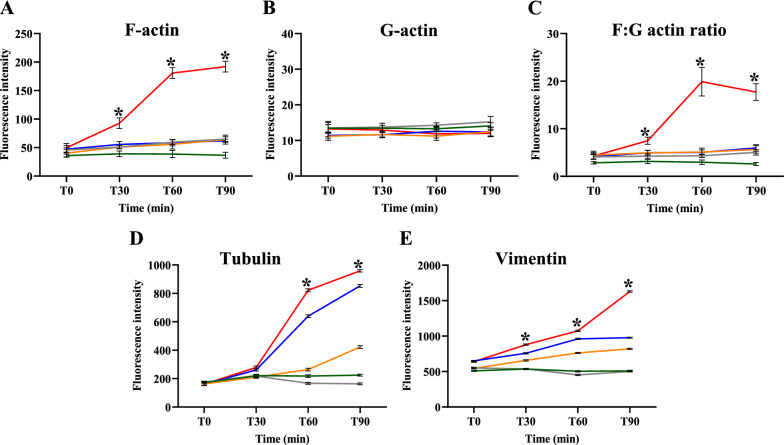
The fluorescence intensity of cytoskeleton in ECs induced by malaria sera. **A**: F-actin; **B**: G-actin; **C**: F:G actin ratio; **D**: tubulin filaments and **E**: vimentin filaments. ECs were incubated with media only (green line), non-malaria patient sera (grey line), malaria sera from *P. vivax* (orange line), uncomplicated *P. falciparum* (blue line) and complicated *P. falciparum* (red line). *Significant difference of complicated *P. falciparum* compared with all experimental groups (*p* < 0.05). Data are presented as mean ± SEM

The accumulated amount of F- and G-actin in the ECs was determined by intensity distribution and fluorescence signals (Alexa 594 phalloidin for F-actin and Alexa Fluor 488 for G-actin). The means phalloidin fluorescence intensity for F-actin at T30, T60 and T90 min in ECs stimulated with malaria sera from complicated *P. falciparum* group were significantly increased when compared with *P. vivax*, uncomplicated *P. falciparum* and control groups (all *p* < 0.05) (Fig. 8A). However, for G-actin, no significant difference in the mean fluorescence intensity was observed at all time points in ECs incubated with sera from complicated *P. falciparum* group compared to all experimental groups (all *p* > 0.05) (Fig. 8B). The fluorescence intensity of F:G actin ratio was highest in ECs stimulated with sera from complicated *P. falciparum* group at all time points when compared with other experimental groups (*p* < 0.05) (Fig. 8C).

#### Tubulin filaments

Tubulin filament accumulation in the ECs was determined by the intensity of fluorescent Alexa Fluor 488. The estimated fluorescence intensity showed significant differences at T60 and T90 min in complicated *P. falciparum* group when compared with all groups (*P. vivax*, uncomplicated *P. falciparum* and control groups) (*p* < 0.05). Figure 8D shows the comparative mean fluorescence intensity of tubulin filaments at different time points.

#### Vimentin intermediate filaments

Vimentin filaments accumulation in the ECs was evaluated by the intensity of fluorescent Alexa Fluor 594. The estimated fluorescence intensity showed a significant difference at T30, T60 and T90 min of complicated *P. falciparum* group when compared with all groups (*P. vivax*, uncomplicated *P. falciparum* and control groups) (Fig. 8E).

### Levels of tumour necrosis factor and interferon-γ

Table 1 demonstrates the levels of TNF and IFN-γ in malaria sera and control groups. TNF and IFN-γ showed similar trends with highest levels detected from complicated *P. falciparum* patients compared to other experimental groups (*p* < 0.05). TNF and IFN-γ levels were 3.8X and 2.4X higher in sera from complicated *P. falciparum* compared to non-malaria patients, respectively.

**Table 1:** Levels of TNF and IFN-γ in malaria sera and control groups

	TNF (pg/ml)	IFN-γ (pg/ml)
Media only	3.43 ± 0.56^a^	4.37 ± 0.92^a^
Non-malaria patient sera	53.13 ± 3.09^a^	40.46 ± 3.10^a^
*P. vivax* sera	103.51 ± 5.49^a^	68.75 ± 3.02^a^
*P. falciparum* (uncomplicated) sera	136.62 ± 8.32^a^	73.75 ± 3.90^a^
*P. falciparum* (complicated) sera	201.51 ± 10.94	98.33 ± 4.84

^a^Significant difference of complicated *P. falciparum* group compared with all experimental groups (*p* < 0.05). n = 4 per experimental group, each performed in triplicate. Data are presented as mean ± SEM

### Correlations between cytoskeleton morphology and fluorescence intensity

**Fig. 9:**
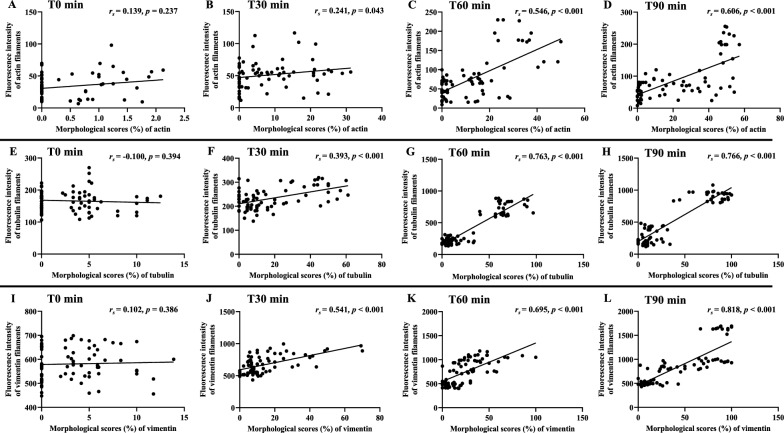
Correlation between morphological changes induced by malaria sera and fluorescence intensity of cytoskeleton at different time points. **A**–**D**: actin filaments; **E**–**H**: tubulin filaments and **I**–**L**: vimentin filaments at different time points

Morphological scores based on overall changes of cytoskeleton filaments and ECs (shrinkage, appearance of eccentric nuclei, presence of “spiking pattern” and fragmentation/discontinuity of actin filaments and ECs) were positively correlated with fluorescence intensity for actin filaments at T60 min (*r*_*s*_ = 0.546, *p* < 0.001) and T90 min (*r*_*s*_ = 0.606*, p* < 0.001) (Fig. 9A–D), for tubulin filaments at T30 min (*r*_*s*_ = 0.393*, p* < 0.001), T60 min (*r*_*s*_ = 0.763*, p* < 0.001) and T90 min (*r*_*s*_ = 0.766*, p* < 0.001) (Fig. 9E–H) and for vimentin filaments at T30 min (*r*_*s*_ = 0.541*, p* < 0.001), T60 min (*r*_*s*_ = 0.695*, p* < 0.001) and T90 min (*r*_*s*_ = 0.818*, p* < 0.001) (Fig. 9I–L).

### Correlations between cytoskeleton morphology and levels of TNF and IFN-γ

**Fig. 10:**
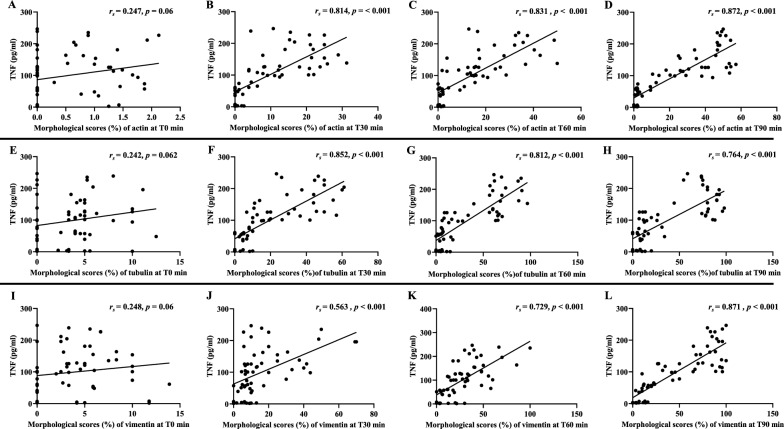
Correlation between morphological changes induced by malaria sera and TNF. **A**–**D**: actin filaments; **E**–**H**: tubulin filaments and **I**–**L**: vimentin filaments at different time points

**Fig. 11:**
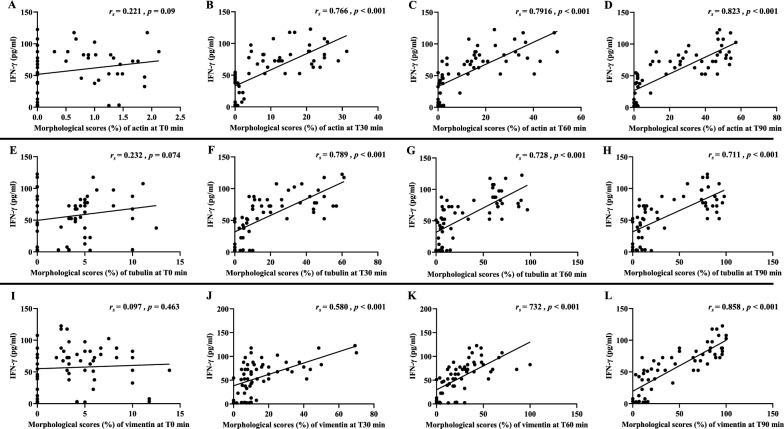
Correlation between morphological changes induced by malaria sera and IFN-γ. **A**–**D**: actin filaments; **E**–**H**: tubulin filaments and **I**–**L**: vimentin filaments at different time points

With regards to the levels of targeted pro-inflammatory cytokines, TNF (Fig. 10) and IFN-γ (Fig. 11) on admission from malaria patients were positively correlated with overall morphological scores of cytoskeleton changes starting from T30 min.

## Discussion

The entire vascular system is lined by ECs, which actively regulate blood flow, maintain blood fluidity, control permeability of water and molecules between vascular lumen and interstitial tissue, and modulate circulating immune cells recruitment and activation [16]. EC barrier is dependent on the complex cytoskeletal network of actin, tubulin and vimentin filaments that are linked to cell junction proteins. ECs play an important role as a sole site of attachment to PRBCs during malaria infection. EC dysfunction is a key feature in malaria pathogenesis leading to impaired blood perfusion, vascular obstruction, and tissue hypoxia [17]. Contributing factors include severe adhesion of PRBCs to vascular ECs, process of sequestration (accumulation of attached PRBCs), rosetting (adhesion between PRBCs and uninfected red blood cells (RBCs), auto-agglutination (adhesion between PRBCs), aggregation (adhesion between uninfected RBCs) and EC activation. The sequelae of cytoadhesion and activation of pro-inflammatory cytokines such as TNF, IL-1 and IFN-γ can cause damage to ECs [3, 18]. EC apoptosis [19, 20] and permeability changes [14] have been reported in post-adhesion process of PRBCs to vascular ECs in severe *P. falciparum* malaria. With the complex interplay of precipitating factors in malaria pathogenesis, both the cytoadhesion and cytokine theories can induce EC activation and initiate the severe sequelae of complicated *P. falciparum* malaria, such as cerebral malaria, pulmonary oedema and acute kidney injury (AKI).

EC barrier integrity is dependent on the intact structures of cytoskeletal proteins (actin, tubulin and vimentin filaments), as well as normal cell junctions. In current study, EC rearrangement and destruction were secondary to cytokines dissolved in malaria sera. Among the three important cytoskeletons in ECs, actin filaments were mostly damaged in ECs induced by sera from complicated *P. falciparum* malaria, considering ranges of alterations. Distinct changes in actin filaments and ECs when stimulated with sera from complicated *P. falciparum* malaria included: (1) shrinkage of actin filaments and ECs with cortical contraction, which leads to EC condensation; (2) focal accumulation of actin filaments near the nuclei, resulting to the appearance of eccentric nuclei; (3) appearance of “spiking pattern” of actin filaments and EC membrane, which suggests redistribution and reorganization of actin filaments towards cell membrane; and (4) fragmentation and discontinuity of actin filaments and ECs, which could alter cytoskeleton rearrangement and EC morphology. All essential morphological changes of actin filaments demonstrated significant damage in ECs incubated with sera from complicated *P. falciparum* malaria, except the findings of fragmentation and discontinuity of actin filaments and ECs, which was minimal in complicated *P. falciparum* group, as majority of actin filaments and ECs had previously undergone shrinkage. From the study, tubulin filament damage was comparable for both uncomplicated and complicated *P. falciparum* malaria groups, suggestive of reduce interaction between tubulin compared to actin filaments, which possess stronger crosslinkers with highly organized and stable actin structures [21]. Vimentin filaments, belonging to intermediate filament group are considered the strongest and most stable cytoskeletal elements [22], and hence illustrated slight disruption in ECs incubated with uncomplicated *P. falciparum* malaria compared to complicated *P. falciparum* malaria group at earlier times (T30 and T60 min).

Of the morphological changes, the appearance of EC shrinkage and presence of eccentric nuclei were the common alterations of the three main cytoskeleton proteins (actin, tubulin and vimentin filaments), which lead to EC damage in ECs treated with sera from complicated *P. falciparum* malaria. The minimal morphological changes observed in ECs treated with sera from *P. vivax* could be related to low levels of TNF and IFN-γ compare to *P. falciparum* malaria, similar to previous study [23]. All alterations indicated ECs undergoing apoptotic process and subsequently EC dysfunction. In malaria infection, studies have reported that pro-inflammatory cytokines such as IL-1, IL-6, IL-8, TNF and IFN-γ were elevated [6, 7, 24]. Cytokines have been demonstrated to induce cytoskeleton changes, such as appearance of “starburst (spiking) patterns” [25], thinning of actin filaments and formation of intercellular gap between actin filaments [26], and tubulin filaments disassembly [27] in ECs incubated with IL-1 [25] and TNF [26, 27]. Actin filament rearrangement can then induce an increase in EC permeability [28] and can cause vascular leakage. Disruption of tubulin filaments has been reported in ECs treated with anti-mitotic agent (colchicine) [29] and the tubulin filament damage was also associated with apoptosis in human colon cancer cell line [30]. In addition, withaferin, a steroidal lactone induced collapsed of vimentin filaments, intercellular gap formation and redistribution of vimentin filaments to the perinuclear region in animal ECs, which could disrupt endothelial barrier functions on vimentin filaments [31].

Pro-inflammatory cytokines, such as TNF, IFN-γ and IL-1, dissolved in sera can cause the activation of transcription nuclear factor kappa B (NF-κB), which subsequently cause expression of other cytokines [32–34]. The stimulated NF-κB signaling cascade has been reported to be correlated with cytoskeleton disorganization [34–36]. In current study, the activated pro-inflammatory cytokines during malaria infection (TNF and IFN-γ) could trigger ECs and result to the disorganization and disruption of cytoskeletal filaments. The damaged cytoskeletal filaments and ECs related to apoptosis could be associated with the induction of NF-κB. In addition, the disruption in cell shape and destabilization of actin, tubulin and vimentin filamentous structures are linked with impaired cell junction, e.g. adherens junctions, tight junctions, and gap junctions [37]. ECs undergo changes in cytoskeletal structures and functions in severe *P. falciparum* malaria can cause the deterioration of EC barrier function and increase in EC permeability. The breakdown of the EC barriers can allow activated cytokines and other harmful substances to enter the tissues and cause life threatening complications, aside from the effect of sequestration.

Fluorescence intensities of F-actin, F:G actin ratio, tubulin and vimentin filaments were highest in ECs stimulated with sera from complicated *P. falciparum* malaria. The increase in fluorescence intensity could be due to the cell shrinkage, contraction and reduction of cell area [35]. The cytoskeleton damage allows increase in fluorescence dye absorption within the filaments. The estimated fluorescence intensity of F:G actin ratio was solely dependent on the intensity of F-actin. As free monomer, G-actin contains lesser surface area and fluorescence absorption could be minimal. The increase in F:G actin ratio determined by protein level has been used as a prognostic factor for sepsis [38]. The results of increase immunofluorescence intensities of cytoskeleton (F-actin, F:G actin ratio, tubulin and vimentin filaments) in ECs stimulated with sera from complication *P. falciparum* malaria could be further investigated for possible prognostic factor for malaria severity. In addition, the overall damages of cytoskeleton and ECs based on morphological scores were positively correlated with the absorbed fluorescence intensity.

## Conclusions

Pro-inflammatory cytokines (TNF and IFN-γ) dissolved in malaria sera have a direct effect on the disorganization of cytoskeleton. Alteration in EC morphology increases fluorescence intensity of F-actin, F:G actin ratio, tubulin and vimentin filaments. Cytoskeleton changes could lead to EC junctional alteration and permeability changes. The findings can serve as a basis to explore measures to strengthen EC cytoskeleton and to reduce EC damage, hence to possibly alleviate severe malaria complications such as pulmonary oedema and cerebral malaria.

## Data Availability

All data generated and analysed during this study are available in the article and the supplementary materials files.

## Abbreviations

AKI: Acute kidney injury
ANOVA: Analysis of variance
℃: Degree Celsius
ECs: Endothelial cells
ELISA: Enzyme-linked immunosorbent assay
F-actin: Filamentous actin
Fig: Figure
G-actin: Globular actin
h: Hour
HRP: Horseradish peroxidase
HUVECs: Human umbilical vein endothelial cells
IFN-γ: Interferon gamma
IL: Interleukin
LSD: Fisher’s least significant difference
min: Minute
ml: Millilitre
n: Number
NF-κB: Nuclear factor kappa B
*p*: *p*-Value
PASW: Predictive Analytics SoftWare
PBS: Phosphate buffered saline solution
PfEMP1: *Plasmodium* *falciparum* Erythrocyte membrane protein 1
pg: Picogram
PRBCs: Parasitized red blood cells
*r*_*s*_: Spearman’s rank correlation coefficient
SEM: Standard error of mean
SPSS: Statistical package for the social sciences
T: Time
TMB: 3, 3’, 5, 5’-Tetramethylbenzidine
TNF: Tumour necrosis factor
